# Challenging Aspects to Precise Health Strategies in Native Hawaiian and Other Pacific Islanders Using Statins

**DOI:** 10.3389/fpubh.2022.799731

**Published:** 2022-02-28

**Authors:** Ligia M. Watanabe, Lucia A. Seale

**Affiliations:** ^1^Division of Nutrition and Metabolism, Department of Health Sciences, Ribeirão Preto Medical School, University of São Paulo, Ribeirão Preto, Brazil; ^2^Pacific Biosciences Research Center, School of Ocean and Earth Science and Technology, University of Hawaii at Mānoa, Honolulu, HI, United States

**Keywords:** Native Hawaiians and Other Pacific Islanders, obesity, statin, genetic variations, cultural values, dyslipidemia

## Abstract

Cardiometabolic disorders (CD), including cardiovascular disease (CVD), diabetes, and obesity, are the leading cause of health concern in the United States (U.S.), disproportionately affecting indigenous populations such a Native Hawaiian and Other Pacific Islanders (NHOPI). Dyslipidemia, a prevalent risk factor for the development and progression of CVD, is more prone to occur in NHOPI than other populations in the U.S. High-intensity statin therapy to reduce low-density lipoprotein cholesterol is associated with the prevention of CVD events. However, significant side-effects, such as muscle disorders, have been associated with its use. Different ethnic groups could experience variation in the prevalence of statin side effects due to sociodemographic, behavioral, and/or biological factors. Therefore, identifying the most impactful determinants that can be modified to prevent or reduce statin side effects for individuals from high-risk ethnic minority groups, such as NHOPI, can lead to more effective strategies to reduce health disparities. Thus, our Mini-Review explores the challenging aspects of public health precise strategies in NHOPI taking statins, including a culturally informed additional therapy that could positively impact the NHOPI population.

## Introduction

The burden of cardiovascular diseases (CVD) and its associated risk factors, such as dyslipidemia, obesity, and diabetes mellitus, are not equally distributed across racial and ethnic groups (1). Data from the National Health Interview Survey shows that Native Hawaiians and Other Pacific Islanders (NHOPI) have a greater prevalence of heart disease relative to non-Hispanic Whites, non-Hispanic Blacks, and Asians (1). Several factors, such as sociodemographic, behavioral, and biological, alone or in combination, may create disparities in health within this population (1, 2).

Dyslipidemia is recognized as a prominent risk factor for CVD (3). In NHOPIs, elevated lipoprotein cholesterol (LDL-C) appeared to potentially impact CVD mortality (4). Thus, studies and current guidelines have focused on aggressive LDL-C lowering with a statin in both primary and secondary intervention settings (2, 4, 5). Nevertheless, significant side effects, such as muscle disorders, have been associated with statin use (6). The occurrence of statin side effects varies among different ethnic groups and is associated with individual intrinsic factors or external aspects (7). Therefore, identifying the most impactful determinants that can be modified to prevent or reduce statin side effects in the NHOPI population is essential to developing interventions that best meet the needs of this population (7).

## General Aspects of Nhopi

NHOPI originated from three ethnogeography regions in Oceania: Melanesia, Micronesia, and Polynesia. These three regions represent 25 Pacific nations (14 sovereign states and 11 collectives) and more than 20,000 islands (8, 9). The largest NHOPI subgroup in the United States, Native Hawaiians, accounts for ~0.4% of the U.S. population (10, 11). NHOPI have the greatest representation in the state of Hawai'i, followed by California, Washington, Texas, Utah, Florida, Nevada, Oregon, New York, and Arizona (2, 5, 10) and are the second-fastest-growing racial/ethnic group in the United States, increasing 40% from 2000 to 2010 (10). Data from the Census Bureau (12) show significant differences in educational attainment, economics, and insurance coverage between NHOPI and non-Hispanic Whites (Table 1) (5), indicating differences in social determinants of health. The World Health Organization (WHO) defines social determinants of health as conditions or circumstances in which people are born, grow, live, work, and age. These conditions are shaped by political, social, and economic forces (13). Social determinants of health directly impact the health of individuals and populations. They also help structure lifestyle choices and behaviors, which interact to produce health or disease (14).

**Table 1:** Social indicators between NHOPI and non-Hispanic Whites according to the Office of Minority Health at U.S. Department of Health and Human Services (5) and Census Bureau (12).

**Social indicator**	**Variables**	**NHOPI**	**Non-Hispanic White**
Educational attainment	High school diplomas or higher	88.7%	93.3%
	Bachelor's degree or higher	23.8%	36.9%
	Graduate or professional degrees	7.4%	14.8%
Economics	Household income	$66,695	$71,664
	Living at the poverty level	14.8%	9%
	Unemployment rate	5.9%	3.7%
Insurance coverage	Private health insurance	65.8%	74.7%
	Medicaid or public health insurance	34%	34.2%
	Uninsured	9.1%	6.3%

*Adapted from Office of Minority Health at U.S. Department of Health and Human Services (5) and Census Bureau (12)*.

Regarding health status, Native Hawaiians display one of the most concerning health profiles and the highest mortality rates for most chronic metabolic diseases when compared to other ethnic groups and the general population in the U.S. (2, 4, 15–17). Also, data from 2000 revealed that Native Hawaiians have the shortest life expectancy rates among all ethnic groups of Hawaii residents (2, 18), evidencing the disproportionate health disparities of this population (5, 17).

## Facets of Cardiometabolic Disorders in Nhopi

The prevalence of cardiometabolic disorders (CD), including CVD, diabetes, and obesity, has reached epidemic proportions worldwide (2). Remarkably, the prevalence of CD among ethnic minorities in the U.S. exceeds those seen in the general population (2, 4, 16, 17), and they are also less likely to receive adequate treatment (17).

Recent national data related to CD for NHOPI is limited (2, 5). Available literature indicated that NHOPI were 10% more likely to be diagnosed, and 68% die of coronary heart disease than the general population (5, 17). Also, Baum et al. (19) assessed national- and state-level geographic variations among patients with a history of ≥ 1 major atherosclerotic CVD (ASCVD) event and found that Hawai'i had the highest proportion of patients with a very high risk of ASCVD events in the U.S (19). Considering the excessive health burden of CD in NHOPI (2), it is urgent to identify the key features of metabolic syndrome in NHOPI to provide specific targets in investigations aiming to reverse and/or eliminate health disparities in this population.

### Dyslipidemia

Dyslipidemia is a powerful and prevalent risk factor for the development and progression of atherosclerosis and CVD (2, 20). Typical dyslipidemia consists of increased triglycerides (TG) and free fatty acids, decreased high-density lipoprotein cholesterol (HDL), and normal or slightly increased low-density lipoprotein cholesterol (LDL) (6). Recently, Sun et al. (11), studying traits for which the Native Hawaiians exhibit excess risk, observed that 2,239 individuals (56.8%) presented hyperlipidemia (or dyslipidemia), in which 657 individuals were undiagnosed. This result corroborates previous findings in which high cholesterol levels were reported by 59% of NHOPI participants, with a higher prevalence among males (66%) compared to females (49%) (15). Together, these findings suggest that a significant part of the NHOPI population may be eligible for statin therapy.

### Obesity

Obesity is one of the central pathophysiologic mechanisms underlying CVDs and presents a major challenge for the health of indigenous populations such as NHOPI (11, 21–24). Obesity in NHOPI contributes to higher mortality and morbidity and a lower expected life span than European or Asian-Americans living in the Hawaiian Islands (11, 22). According to the Centers for Disease Control and Prevention (CDC), in 2016, NHOPI were three times more likely to have obesity than the overall Asian-American population and 80% more than non-Hispanic Whites (25). Indeed, epidemiological studies have shown that 49% of Native Hawaiians present with obesity (11), and 34% have overweight (22), resulting in concurrent high rates of obesity-related pathologies, such as dyslipidemia and CVD (22).

### Diabetes

The Native Hawaiian Health Research (NHHR) Project examined the relationship between the clustering of cardiovascular risk factors and biochemical markers of insulin resistance (2). The investigators found that fasting insulin concentrations were correlated with body mass index, waist-to-hip ratio, blood pressure, and levels of TG, LDL, and glucose. A significant correlation was also found between increasing insulin resistance and increased cardiovascular disease risk factors (2).

Alarmingly, the prevalence of diagnosed diabetes in Native Hawaiians is the highest among United States populations, ranging from 19 to 22% for type 2 diabetes and from 16 to 35% for impaired glucose tolerance (2). Moreover, recent results from the study of Sun et al. (11) alerted a high frequency of undiagnosed type 2 diabetes in Native Hawaiians, as they found 1,799 (57.9%) of 3,109 individuals with undiagnosed type 2 diabetes.

## Facets of Statin Therapy in Nhopi

Cholesterol-lowering therapy, with low-density lipoprotein cholesterol (LDL-C) as a primary target, is beneficial in preventing CVD (26). Statins are a widely effective pharmacological therapy for treating hypercholesterolemia and preventing cardiovascular events, prescribed mainly due to their safety profile (27). There are currently seven types of statins approved by the U.S. Food and Drug Administration (FDA): atorvastatin, fluvastatin, lovastatin, pitavastatin, pravastatin, rosuvastatin, and simvastatin (28). Except for pravastatin, statins are metabolized by the cytochrome P450 pathway, transforming lipophilic compounds into hydrophobic in the liver for excretion (29, 30). Atorvastatin, lovastatin, and simvastatin are metabolized primarily by cytochrome P450 3A4 (CYP3A4) (30–32). Medications also metabolized by CYP3A4 can interact with statins, increasing the risk of side effects (30, 31, 33). Other statins, such as fluvastatin, pitavastatin, and rosuvastatin, are minimally metabolized by CYP2C9 and have fewer statin–drug interactions (30–32).

The 2018 American College of Cardiology/American Heart Association guideline recommends high-intensity statin doses for individuals presenting one or more risk factors, such as age ≥65 years, family history of premature ASCVD, primary hypercholesterolemia, metabolic syndrome, chronic kidney disease, chronic inflammatory conditions, history of premature menopause (before age 40) and of pregnancy-associated conditions that increase later ASCVD risk such as pre-eclampsia, high-risk ethnicities, with dysregulated lipid/biomarkers associated with increased ASCVD risk, and currently smoking (34, 35). The guideline also considered diabetes-specific risk enhancers for the recommendation of statin therapy, such as long duration (≥10 years for type 2 diabetes or ≥20 years for type 1 diabetes), albuminuria ≥30 mcg albumin/mg creatinine, glomerular filtration rate <60 ml/min/1.73 m^2^, retinopathy, neuropathy, and ankle-brachial index <0.9 (34).

Considering the health indicators of NHOPI, especially the elevated rates of metabolic syndrome, diabetes, dyslipidemia, and smoking (5, 15), they could be classified as a higher-risk population in which the high-intensity statin doses are recommended (5). Indeed, the statin safety recommendations for NHOPI in the 2018 Guideline on the Management of Blood Cholesterol of ACC follow the Hispanics/Latinos population in the U.S. and include Whites, Blacks, and Native Americans (34), although the pharmacokinetics in NHOPI is understudied (11). Thus, the Food and Drug Administration (FDA) recommended a starting dose of 10 mg of rosuvastatin for Whites vs. a lower dose for Asians, 5 mg (34).

Overwhelmingly, muscle-related symptoms are the most reported side effect among statin-treated patients, affecting ~10 to 29% of patients and contributing to statin therapy non-adherence or discontinuation (35). However, the number of patients suffering from statin myalgia and other mild muscle-related symptoms may be higher, primarily due to the absence of definitive diagnostic tests (36). The current evaluation of statin muscle-related symptoms is based on clinical criteria (patient self-reported description of symptoms) and non-invasive diagnostic tools, such as statin challenge-rechallenge protocol, creatine kinase (CK), Statin-Associated Muscle Symptoms Clinical Index questionnaire, American College of Cardiology statin intolerance tool, and indirect measurements of mitochondrial function (36). The high prevalence of statin uses by the U.S. population, combined with current guidelines recommending high-intensity statins to obtain the lowest LDL-C levels for many patients, warns of statin adverse side effects. Therefore, clarifying contributing factors to statin response variability may be an important public health accomplishment (35, 37).

Discerning effects of several lipid-lowering drugs can be seen in different ethnic groups because of variations in genetic background, sex, drug metabolism, and environmental factors, such as diet, stress levels, alcohol consumption, and medication adherence (7). Differences in statin blood levels, pharmacokinetics, and their efficacy and adverse effects are known to be caused by genetic variations among populations (7, 38). Potential genetic variations have been linked to differential response to statin and statin-related side effects, explaining ethnicity as a source of response variability observed between Asians and Americans. For example, the ATP-binding cassette, subfamily G, member 2 (ABCG2) polymorphism (rs2231142) is more common in Asians than Americans, contributing to higher statin concentration in plasma and lower lipid response to statins. Higher prevalence of polymorphisms rs4363657 C and rs4149056 C alleles in Solute Carrier Organic Anion Transporter Family Member 1B1 (SLCO1B1) in Asians have been associated with reduced hepatic uptake of statins, resulting in higher circulating statin concentrations and an increased risk of myopathies. The authors suggested that SCLO1B1 polymorphisms increased circulating statin levels due to effects that lead to less toxicity to muscle cells for hydrophilic agents, such as rosuvastatin and pravastatin, compared with more hydrophobic statins such as simvastatin (38).

Interestingly, Lum et al. (39) demonstrated that simvastatin was the most prescribed statin in Hawai'i in 2013 and had a lower cost per day ($0.25/day) than rosuvastatin ($5.53/day). On the other hand, simvastatin is one of the most returned medications in drug take-back events held by the Hawai'i Narcotics Enforcement Division (NED) of the Department of Public Safety and the University of Hawai'i at Hilo Daniel K. Inouye College of Pharmacy on four major islands in 2011 and 2012 (40). From this information, it would be critical to investigate the reasons for the high rate of return of simvastatin in the Hawaiian population since its use is continuous. One possibility would be that the adverse effects connected to the use of simvastatin prevail for this drug return. In the general population, statin-associated muscle symptoms are the primary reason for discontinuation and non-adherence to statin therapy and are more common with simvastatin than other available statins (27). It also remains to be investigated if NHOPI may present a particular genetic polymorphism that may enhance or protect against statin-associated muscle symptoms in this population.

## Discussion

Although the health disparities of the NHOPI are well-recognized, scientific data on this population is limited and often masked by the misperception of considering NHOPI in the same ethnic group as Asian-Americans (24). The available information indicated that the NHOPI are more likely to develop obesity and associated diseases, such as dyslipidemia, either by genetic risk profile or the insertion in a context of deconstruction of cultural habits, especially to food consumption patterns, which further contribute to high rates of chronic diseases in this population. Considering the high use of statins and the potential acute side effects associated with their use, especially muscle toxicity, the possible prevalence of polymorphisms in genes related to different responses to statins and statin adverse effects in NHOPI emphasize the importance of considering the ethnicity to adjust medication prescription and avoid adverse health outcomes.

Disease prevention, treatment, and management programs must be grounded in cultural values at their core and the cornerstone of health promotion. Health programs aligned with cultural values, perspectives, and preferred modes of living of an indigenous population are known as culturally responsive programs (8). Along with community and cultural informants in community-based participatory research settings, investigators demonstrated that Polynesian dances, such as the well-known Hawaiian Hula, can be used effectively and appropriately as part of health interventions (16, 41). Hula, the traditional dance of Hawai'i and its indigenous people, preserves significant aspects of Native Hawaiian culture, with strong ties to health and spirituality (16, 17).

Hula comprises specific controlled rhythmic movements that vary in intensity and duration, depending on the choreography (16, 17). Several features make Hula a viable strategy to prevent CVD, improving overall health. Specifically, the energy expenditure of Hula yields a metabolic equivalent (MET) of 5.7 for moderate-intensity and 7.6 for high-intensity forms (17). This range of MET and oxygen uptake values are comparable to doubles tennis (7.0), brisk walking (4.0), swimming at a moderate level (8.0), and a basketball game (8.0) (42). Hula could also be effective for cardiac rehabilitation, as it has low intensity, is prolonged, and can achieve 70 to 80% of the maximal predicted heart rate if pursued three times a week (43). Recently, a randomized controlled trial tested the effects of a 6-month cultural dance program based on Hula for improving blood pressure (BP) and CVD risk among Native Hawaiians with uncontrolled higher hypertension. They found that the intervention yielded more significant reductions in systolic (−15.3 mmHg) and diastolic (−6.4 mmHg) BP than control (−11.8 and −2.6 mmHg, respectively) from baseline to 6 months (*p* < 0.05). Also, the 10-year CVD percent risk reduction was greater for the intervention group (−6.4%) than the control group (−2.8%) based on the Framingham Risk Score—cardiovascular diseases calculator. All improvements for intervention participants were maintained at 12 months (44). Transcending the physical activity aspect, Hula also incorporates the integrated Hawaiian values such as familial relationships, cooperation, and aloha—ongoing kindness and acceptance of others (8).

The impressive impact of Hula practice on cardiovascular and associated events could be helpful as adjunctive therapy to medications such as statins. Considering the health benefits of Hula, its addition as part of lifestyle changes in a combination of a low-dose or an intermittent dosing statin may allow patients to achieve their lipid goals while limiting toxicity from the drug therapy. More studies are needed to investigate this possibility in NHOPI and better understand the combined impact between Hula practice and statin dosage.

## Conclusion

In conclusion, identifying aspects that influence the occurrence of CVD and its associated risk factors, such as dyslipidemia, and the impact caused by genetic variations in statin side effects in NHOPI, are the first steps in understanding the broad way in which sociodemographic, behavioral, and biological factors may be associated with chronic illness risk in this population. Also, we highlight the importance of a culturally-based intervention for high-risk ethnic populations to “close the gap” in health disparities and create alignment with their cultural values and preferences, making them more relevant, accessible, and sustainable.

## Author Contributions

LW and LS: conceived, wrote, read, and approved the final manuscript.

## Funding

The authors are funded by grant numbers 2020/08687-9 from Fundação de Amparo a Pesquisa do Estado de São Paulo (FAPESP) to LW; R01DK128390-01 from the National Institute of Diabetes and Digestive and Kidney Diseases (NIDDK) and 20ADVC-102166 from the Hawaii Community Foundation to LS.

## Conflict of Interest

The authors declare that the research was conducted in the absence of any commercial or financial relationships that could be construed as a potential conflict of interest.

## Publisher's Note

All claims expressed in this article are solely those of the authors and do not necessarily represent those of their affiliated organizations, or those of the publisher, the editors and the reviewers. Any product that may be evaluated in this article, or claim that may be made by its manufacturer, is not guaranteed or endorsed by the publisher.
